# Subjective well-being among young people in five Eastern European countries

**DOI:** 10.1017/gmh.2017.8

**Published:** 2017-07-06

**Authors:** M. S. C. Lim, C. Cappa, G. C. Patton

**Affiliations:** 1 Burnet Institute, Centre for Population Health, 85 Commercial Road, Melbourne 3004, Australia; 2 Monash University, School of Population Health and Preventive Medicine, 89 Commercial Road, Melbourne 3004, Australia; 3 UNICEF, Data and Analytics Section, Division of Data, Research and Policy (DRP), 3 UN Plaza, New York 10017, USA; 4 Murdoch Children's Research Institute, Royal Children's Hospital, Flemington Road, Parkville 3052, Australia

**Keywords:** Eastern Europe, happiness, life satisfaction, optimism, subjective well-being, young people

## Abstract

**Background.:**

Subjective well-being incorporates elements of life satisfaction, happiness and optimism. It is increasingly relevant in the assessment of population health and economic development. There are strong continuities in well-being from youth into later life. Despite its significance, few global surveys capture subjective well-being. This paper describes patterns of well-being among young people in five Eastern European countries [Belarus, Bosnia and Herzegovina (BiH), the Former Yugoslav Republic of Macedonia, Serbia and Ukraine] and investigates association between demographic factors and well-being.

**Methods.:**

Nationally representative household surveys, including large Roma population samples, were conducted as part of UNICEF's Multiple Indicator Cluster Survey programme. Young people aged 15–24 years (*N* = 11 944) indicated their satisfaction with life, happiness and expectations about the future. Multilevel logistic regressions were conducted to determine the impact of individual-level predictors while accounting for country- and cluster-level variability.

**Results.:**

Around 40% of young people considered themselves very happy or very satisfied with their life overall. Three quarters reported optimism. Yet well-being varied greatly between countries, with youth in BiH and Ukraine reporting lowest levels of well-being. Current marriage, increasing wealth, higher education, rural residence and not having children were associated with greater well-being.

**Conclusions.:**

Patterns of well-being in youth vary substantially between countries and are only partly accounted for by standard demographic characteristics. Despite higher rates of adolescent marriage and childbearing, and lower levels of educational attainment and employment, Roma youth had similar levels of well-being to the general population.

## Introduction

Economic development has brought many health benefits in low- and middle-income countries (LMIC). These include lower maternal and infant deaths, and lower rates of infectious diseases through childhood and adolescence. Concurrently, patterns of health and health risks have shifted, with higher suicide mortality, injury and non-communicable diseases among adolescents and young adults (Patel *et al.* 2012; Patton *et al.* 2012). This has raised questions about the effect of economic development on patterns of mental health and subjective well-being. In high-income countries, subjective well-being has fallen over the past century, accompanied by rising rates of mental disorders (Prosser & McArdle, 1996; Twenge, 2011). For these reasons subjective well-being is increasingly recognised as a relevant measure of population health (Prince *et al.* 2007; Dolan *et al.* 2008) and some scholars have argued that measures of subjective well-being are better measures of societal progress than traditional measures such as gross domestic product (GDP) (Stiglitz *et al.* 2008).

Subjective well-being refers to a person's self-evaluation of their own life. Definitions used in research vary, but include measures of satisfaction with life, positive mood and affect, and absence of negative mood and affect (Diener, 1994; Snyder & Lopez, 2002; Diener *et al.* 2017). Well-being can be considered a global measure incorporating positive subjective evaluation of life satisfaction (past), positive emotions such as happiness (present), and optimism (future) (Snyder & Lopez, 2002). Research has shown that these different components each make a separate but important contribution to overall subjective well-being, and that it is therefore important to measure multiple dimensions to form a complete picture (Diener *et al.* 2017). In this study, we measure subjective well-being using the three constructs of overall life satisfaction, happiness and optimism.

Subjective well-being can be assessed both globally and individually. There is a strong positive association between a country's GDP and its collective level of subjective well-being, with wealthier countries demonstrating higher subjective well-being (Deaton, 2008; Levin *et al.* 2011). Between 2012 and 2014, the Gallup World Poll surveyed respondents aged 15 and older from 156 countries, measuring responses to a life satisfaction question on an 11-point scale from 0 (‘the worst possible life’) to 10 (‘the best possible life’) (Helliwell *et al.* 2015). It was found that Western European countries, plus Canada, New Zealand and Australia, had the highest levels of life satisfaction, scoring over 7.2 out of 10. Sub-Saharan African countries, Syria and Afghanistan were the least satisfied, scoring below 3.7 on average. The Gallup Organization also collected data in 2013 using the Global Well-Being Index, which classifies responses as ‘thriving’, ‘struggling’ or ‘suffering’ across purpose, social, financial, community and physical well-being domains. Gallup found that participants (aged 15 and older) in Latin America and Europe were most likely to be ‘thriving’ and sub-Saharan Africa was the least ‘thriving’ region (Standish & Witters, 2014). At an individual level, income is strongly associated with subjective well-being in LMIC, but in wealthier countries, personal income and subjective well-being are not closely related (Ventegodt *et al.* 2008; Levin *et al.* 2011; Yiengprugsawan *et al.* 2012). One reason may be that once an individual achieves an income level that allows for basic needs to be met, further increases in income have minimal impact on subjective well-being (Diener, 2002; Levin *et al.* 2011). Inequality in relative income, rather than absolute income, may better predict subjective well-being in these settings (Dolan *et al.* 2008; Levin *et al.* 2011).

It is particularly important to study the subjective wellbeing of young people in various settings. Youth is a time of rapid change when life-course trajectories of health and well-being are established; subjective well-being in adolescence has been shown to influence health and behaviour patterns that persist throughout adulthood (Patton *et al.* 2011; Currie *et al.* 2012). Poor life satisfaction among youth has been associated with increased risk-taking behaviour, violence, substance abuse, sexual risk behaviours and poor diet, all of which can lead to negative health outcomes (Proctor *et al.* 2009). Youth happiness is also strongly linked to mental health and adult life satisfaction (Proctor *et al.* 2009; Jewell & Kambhampati, 2015). The ages between 15 and 24 years represent a period of transition between childhood and adulthood, including growing independence, transitions between schooling and employment, and for many, marriage and children. Rapid social, economic and political change is likely to affect youth well-being. Surveys in 1999–2002 and 2006 found that countries in the Eastern European region had some of the lowest levels of well-being in the world (Tov & Diener, 2007; Deaton, 2008).

Statistics on subjective well-being remain relatively rare, particularly for LMICs and among young people (Proctor *et al.* 2009; Goldin, 2014). Few studies have measured all three areas of subjective well-being (happiness, optimism and life satisfaction); for example, in a large systematic review by Dolan *et al.* (2008), none of the 19 major datasets identified included questions on all three areas.

This paper describes research on subjective well-being among young people in five Eastern European countries: Belarus, Bosnia and Herzegovina [BiH], the Former Yugoslav Republic of Macedonia [FYRoM], Serbia and Ukraine, including large samples of Roma youth. It was designed to determine the association between individual-level demographic factors and life satisfaction, happiness and optimism, controlling for country-level variability.

## Methods

### Data sources

The study was based on data from five UNICEF-supported Multiple Indicator Cluster Surveys (MICS) conducted between 2010 and 2012 in Belarus, BiH, FYRoM, Serbia and Ukraine (UNICEF, 2015). The surveys included a nationally representative sample of youth aged 15–24 years in each of the five countries, and separate representative samples of youth aged 15–24 living in Roma settlements in three of the five countries (BiH, FYRoM and Serbia).

MICS are household surveys that produce statistically sound, internationally comparable estimates of socioeconomic and health indicators. Since its inception in the mid-1990s, this international household survey programme has enabled more than 100 LMICs to collect nationally representative and internationally comparable data on more than 100 key indicators in areas such as nutrition, child health, mortality, education, water and sanitation, child protection and HIV/AIDS. To date, five rounds of MICS have been completed (MICS1: 1995–1996; MICS2: 2000–2001; MICS3: 2005–2006; MICS4: 2009–2012; MICS5: 2013–2016), and a sixth round is in progress.

The MICS survey tools are developed by UNICEF in consultation with relevant experts from various UN organizations and interagency monitoring groups. The core tools include a household questionnaire, a questionnaire for individual girls and women between the ages of 15 and 49, and a questionnaire on children under age five (administered to the mothers or primary caregivers). An individual men's questionnaire has been administered since MICS4. The questionnaires are all modular in nature and can be adapted or customized to the needs of the country.

The fourth round of MICS (MICS4) included a new optional module on subjective well-being for young people aged 15–24 years. The module was developed by UNICEF for the purposes of MICS. For analysis and ease of interpretation, outcomes were dichotomized into higher and lower well-being categories, a strategy that has been used in other well-being research (Levin *et al.* 2011). The module asks about satisfaction with life overall, with response options of ‘very satisfied’, ‘somewhat satisfied’, ‘neither satisfied nor unsatisfied’, ‘somewhat unsatisfied’ and ‘very unsatisfied’; responses were dichotomized as ‘very satisfied’ *v.* all other responses. Participants were asked how happy they were on a five-point scale ranging from ‘very happy’ to ‘very unhappy’; responses were dichotomized as ‘very happy’ *v.* all other responses. To assess optimism, participants were asked ‘In one year from now, do you expect that your life will be better, will be more or less the same, or will be worse, overall?’; responses were dichotomized as ‘expect life will be better’ *v.* all other responses.

### Sample

The surveys were based on nationally representative samples of households. To allow for accurate estimation of major indicators, households with children under 5 years old were oversampled. Roma households were surveyed using a separate sampling frame in BiH, FYRoM and Serbia.

The urban and rural domains within geographic areas were identified as the main sampling strata and the sample was selected in two stages. Within each stratum, a specified number of census enumeration areas (clusters) were selected systematically with probability proportional to size. After a household listing was carried out within the selected clusters, the listed households were divided into households with and without children under 5, and a separate systematic sample of households was selected for each group. Roma households, identified from past censuses, were also randomly selected from a stratified one-stage sample.

Sampling weights were applied to the data to represent national census data using:

- probability of household selection within a cluster (i.e. the reciprocal of the sampling fraction within the cluster), determined by the number of households in a cluster; and
- non-response rate within the cluster.

Sampling methodology for each country is described in depth in MICS country-specific reports (UNICEF, 2015).

### Analysis

Multilevel logistic regressions were conducted to determine the impact of individual-level predictors on measures of subjective well-being, accounting for country- and cluster-level variability. Applying random effects for countries accounted for variability between countries and excluded any unmeasured differences between countries, permitting examination of the effects of all the other (fixed) variables. The data were formatted hierarchically in three levels (country, cluster and individual). Country and cluster were treated as nested random factors to account for the variable effects of these on individual-level predictors. Three outcome variables were modelled: happiness, life satisfaction and optimism.

Demographic variables included in analysis were the sex of the respondents (male and female), age group (15–19 years or 20–24 years), ethnicity (Roma or non-Roma), household wealth (in quintiles determined using principal components analysis of assets, dwelling characteristics, water and sanitation), place of residence (urban and rural households), highest level of education achieved, marital status, having ever had any children, age at marriage (<18 years or 18 years and older), and self-reported employment, income and current participation in education. Having children was not included in the main analysis as this question was not asked of men. Similarly, age at marriage was not included in the main analysis as this question was only asked of participants who had ever been married. However, the analysis was repeated with women to see if different levels of well-being were reported among women with children, and with ever-married participants to explore the association between well-being and marital status. Chi2 tests, accounting for sample weights, were used to determine differences in demographic characteristics between men and women and between Roma and general population young people.

## Results

### Participants

Household response rates ranged from 86% among Roma settlements in BiH to 98% in Belarus. Across five countries there were 11 944 participants aged 15–24 years, including 7942 (66%) females and 4002 (34%) males. One quarter (2827, 24%) of the sample were Roma.

Demographic characteristics are presented in Table 1. There were statistically significant differences between Roma and non-Roma respondents in terms of current employment, current income, current study, marital status, ever having had children, wealth quintile and completion of tertiary studies. For example, 40% of male and 62% of female Roma were currently or previously married, compared with 9% of males and 25% of females in the general population samples. Just 1% of Roma had completed tertiary education (*v.* 37% of non-Roma), less than one quarter had completed secondary school or higher education (*v.* 95% of non-Roma), and only 17% were currently enrolled in any schooling (*v.* 59% of non-Roma). Half of Roma women had children, compared with 17% of non-Roma women.

**Table 1.** Demographics and measures of subjective well-being by gender and Roma ethnicity, of participants aged 15–24 (weighted percentages)

	General population			Roma Settlements			All		
	Women	Men	*All*	Women	Men	*All*	Women	Men	*All*
Age**									
15–19	45.2	46.2	*45*.*5*	51.7	50.6	*51*.*2*	46.5	47.2	*46*.*8*
20–24	54.8	53.8	*54.5*	48.3	49.4	*48.8*	53.5	52.8	*53.2*
Marital status*^,^**									
Currently married	23.3	8.4	*18.1*	55.2	36.5	*47.4*	29.6	15.2	*24.3*
Previously married	1.8	0.6	*1.4*	6.6	3.9	*5.4*	2.7	1.4	*2.3*
Never married	74.9	90.9	*80.5*	38.2	59.6	*47.1*	67.7	83.4	*73.4*
Age at marriage^a^*^,^**									
<18 years	18.3	9.8	*16.9*	71.2	47.7	*63.7*	38.1	32.0	*36.7*
≥18 years	81.7	90.2	*83.1*	28.8	52.3	*36.3*	61.9	68.0	*63.3*
Ever had child(ren)^b^**									
Yes	17.1	–	*17.1*	49.8	–	*49.8*	23.5	–	*23.5*
No	82.9	–	*82.9*	50.2	–	*50.2*	76.5	–	*76.5*
Household wealth quintile**									
Poorest	15.4	15.1	*15.3*	20.6	19.3	*20.1*	16.4	16.1	*16.3*
Second	20.6	18.3	*19.8*	19.4	18.8	*19.2*	20.4	18.4	*19.7*
Middle	19.5	22.3	*20.5*	20.3	21.3	*20.7*	19.7	22.1	*20.6*
Fourth	21.9	21.4	*21.7*	19.3	21.6	*20.2*	21.4	21.5	*21.4*
Richest	22.5	22.9	*22.6*	20.3	19.0	*19.8*	22.1	21.9	*22.0*
Region*									
Urban	59.4	54.0	*57.5*	57.8	41.0	*50.8*	59.1	50.9	*56.1*
Rural	40.6	46.0	*42.5*	42.2	59.0	*49.2*	40.9	49.1	*43.9*
Highest education*^,^**									
None	0.1	0.0	*0.1*	16.9	10.1	*14.1*	3.4	2.5	*3.1*
Primary	5.8	3.8	*5.1*	63.2	63.0	*63.1*	17.1	18.0	*17.4*
Secondary	53.5	65.1	*57.6*	18.5	26.5	*21.8*	46.6	55.8	*50.0*
Higher	40.5	31.1	*37.2*	1.4	0.4	*1.0*	32.9	23.7	*29.5*
Currently studying*^,^**									
Yes	60.4	55.9	*58.8*	15.4	18.5	*16.7*	51.6	46.9	*49.9*
No	39.6	44.1	*41.2*	84.6	81.5	*83.3*	48.4	53.1	*50.1*
Have job*^,^**									
Yes	22.8	29.5	*25.1*	9.0	27.5	*16.7*	20.1	29.0	*23.4*
No	77.2	70.5	*74.9*	91.0	72.5	*83.3*	79.9	71.0	*76.6*
Have income									
Yes	38.8	40.4	*39.4*	33.5	38.9	*35.7*	37.8	40.0	*38.6*
No	61.2	59.6	*60.6*	66.5	61.1	*64.3*	62.2	60.0	*61.4*
Subjective well-being									
Very satisfied overall	43.3	36.5	*40.9*	44.1	37.0	*40.9*	43.5	36.6	*40.9*
Very happy	40.5	30.6	*37.0*	46.0	41.2	*44.0*	41.6	33.2	*38.5*
Optimistic	75.5	70.6	*73.8*	78.1	69.0	*74.4*	76.1	70.2	*73.9*

a Only asked of those ever married.

b Only asked of women.

*Significant difference in percentage between men and women (*p* < 0.05)

**Significant difference in percentage between Roma and general population (*p* < 0.05)

In the sample overall, females were statistically significantly more likely than males to live in urban areas (59% *v.* 54%), to have completed tertiary education (33% *v.* 24%) and to be currently studying (60% *v.* 56%), but were less likely to be currently employed (20% *v.* 29%) (Table 1). Females were statistically significantly more likely than males to be currently married (30% *v.* 15%) and to have been married before the age of 18 years (38% *v.* 32% among those ever married). Some gender differences were greater among Roma populations, including employment (9% of Roma women were employed *v.* 28% of Roma men, *p* *<* 0.01), and marriage before the age of 18 years (71% of ever-married Roma women *v.* 48% of Roma men, *p* *<* *0.01*).

### Correlates of well-being

Measures of subjective well-being varied between countries and populations (Table 2). Respondents in BiH and Ukraine generally demonstrated poorer subjective well-being than respondents in Belarus, FYRoM and Serbia. A broad range of social and contextual factors were associated with the different levels of well-being, including wealth status, education, employment, marital status and parenthood.

**Table 2.** Measures of subjective well-being, by country, gender and Roma ethnicity, of participants aged 15–24 (weighted percentages)

			Very satisfied overall	Very happy	Optimistic
Belarus	All	Women	50.4	46.5	85.6
		Men	52.2	40.5	80.9
	*Total*		*50.9*	*44.8*	*84.3*
Bosnia and Herzegovina	General population	Women	20.1	19.7	79.4
		Men	24.6	22.5	74.7
	Roma settlements	Women	18.4	23.5	76.5
		Men	23.6	29.6	60.5
	*Total*		*22.1*	*22.8*	*74.4*
Former Yugoslav Republic of Macedonia	General population	Women	65.2	57.3	83.6
	Roma settlements	Women	56.7	53.9	74.5
	*Total*		*63.1*	*56.5*	*81.3*
Serbia	General population	Women	59.7	60.9	83.3
		Men	55.3	47.1	75.8
	Roma settlements	Women	55.2	57.0	80.9
		Men	50.3	52.6	77.5
	*Total*		*56.1*	*55.1*	*79.9*
Ukraine	All	Women	30.0	26.1	55.2
		Men	25.0	19.2	50.9
	*Total*		*28.5*	*24.0*	*53.9*

### Life satisfaction

Overall, 41% of respondents were very satisfied with their life overall (Table 1).

Table 3 shows correlates of being very satisfied with life in multilevel analysis. Odds of being very satisfied with life were 29% lower in those aged 20–24 than in those aged 15–19 years. Sex and Roma ethnicity were not significantly associated with life satisfaction. Those who were currently married were 79% more likely to report being very satisfied than those who were never married, who in turn were nearly twice as likely to report being very satisfied as those who had been previously married. Among those who had ever been married, marriage before age 18 years was not significantly associated with life satisfaction. Women who had children were 30% less likely to report being very satisfied than women without children.

**Table 3.** Proportion very happy, very satisfied, and optimistic, by demographic characteristics; and association between demographic factors and subjective well-being in multi-level model

	*n*	% very satisfied	Adjusted odds ratios (95%CI)	% very happy	Adjusted odds ratios (95%CI)	% optimistic	Adjusted odds ratios (95%CI)
Gender							
Male	4002	36.6	1.0	33.2	1.0	70.2	1.0
Female	7942	43.5	0.92 (0.83–1.02)	41.6	1.08 (0.97–1.19)	76.1	1.43 (1.28–1.60)
Age							
15–19	4995	43.6	1.0	41.5	1.0	74.6	1.0
20–24	6949	38.6	0.71 (0.63–0.80)	35.8	0.64 (0.57–0.72)	73.4	0.81 (0.71–0.92)
Ethnicity							
Non-Roma	9117	40.9	1.0	37.0	1.0	73.8	1.0
Roma	2827	41.2	0.91 (0.75–1.11)	44.0	1.04 (0.86–1.25)	74.4	1.13 (0.91–1.41)
Marital status							
Currently married	4242	46.5	1.79 (1.58–2.01)	48.7	2.34 (2.07–2.64)	74.3	1.12 (0.99–1.28)
Previously married	384	20.9	0.52 (0.39–0.71)	15.4	0.43 (0.31–0.59)	65.2	0.96 (0.74–1.26)
Never married	7318	39.7	1.0	35.8	1.0	74.1	1.0
Age at marriage^a^							
<18 years	1622	42.4	0.88 (0.73–1.05)	44.9	0.71 (0.59–0.85)	71.7	1.01 (0.83–1.23)
≥18 years	3004	43.8	1.0	46.5	1.0	74.6	1.0
Have child(ren)^b^							
Yes	3215	42.9	0.70 (0.57–0.86)	44.7	0.84 (0.69–1.03)	71.7	0.58 (0.46–0.73)
No	4727	45.1	1.0	40.6	1.0	77.4	1.0
Household wealth quintile			1.26 (1.21–1.31)		1.25 (1.20–1.30)		1.13 (1.09–1.18)
Poorest	2386	32.2		32.7		70.3	
Second	2522	40.5		37.4		72.2	
Middle	2351	40.8		36.3		72.8	
Fourth	2355	42.9		41.0		75.2	
Richest	2330	46.1		43.4		77.9	
Region							
Urban	6237	43.9	1.0	40.8	1.0	73.7	1.0
Rural	5707	37.1	1.33 (1.16–1.51)	35.5	1.34 (1.17–1.52)	74.2	1.16 (1.01–1.35)
Highest education							
None/ Primary	2911	41.4	1.0	44.2	1.0	73.1	1.0
Secondary	5947	40.0	1.03 (0.88–1.22)	37.5	1.06 (0.91–1.26)	74.2	1.49 (1.25–1.79)
Higher	3082	42.3	1.25 (1.01–1.55)	36.2	1.29 (1.04–1.59)	74.1	2.17 (1.72–2.74)
Currently studying							
Yes	4895	45.4	1.53 (1.34–1.75)	40.4	1.30 (1.14–1.48)	76.3	1.16 (1.00–1.34)
No	7036	36.6	1.0	36.6	1.0	71.6	1.0
Have job							
Yes	2909	39.0	1.17 (1.02–1.35)	35.4	1.11 (0.97–1.28)	71.7	0.95 (0.82–1.11)
No	9035	41.5	1.0	39.4	1.0	74.6	1.0
Have income							
Yes	5020	38.8	0.98 (0.86–1.11)	36.3	0.97 (0.86–1.10)	73.3	1.22 (1.07–1.40)
No	6924	42.3	1.0	39.8	1.0	74.3	1.0

a Only asked of those ever married.

b Only asked of women.

Each quintile increase in household wealth was associated with a 26% increase in odds of being very satisfied with life. Those residing in rural areas were 33% more likely to report being very satisfied. Compared with primary level education, tertiary education was associated with 25% greater odds of being very satisfied with life. Current studying and current employment were also associated with increased odds (53 and 17%, respectively) of reporting being very satisfied. A current personal income was not associated with life satisfaction.

### Happiness

Overall, 39% of respondents reported feeling very happy (Table 1).

Table 3 shows correlates of being very happy in multilevel analysis. Odds of being very happy were 36% lower in those aged 20–24 than 15–19 years. Roma participants and female participants were slightly more likely to report being very happy, but this difference was not statistically significant when adjusted for other factors (Table 3). Those who were currently married were more than twice as likely to report being very happy as those never married, who in turn were more than twice as likely to report being very happy than those who had been previously married. Among those ever married, marriage before age 18 years was associated with a 29% decrease in happiness. Among women, having children was not significantly associated with happiness.

Each quintile increase in household wealth was associated with a 25% increase in odds of being very happy. Those residing in rural areas were 34% more likely to report being very happy. Compared with primary level education, tertiary education was associated with 29% greater odds of being very satisfied with life. Current studying was associated with a 30% increase in odds of being very happy. Current employment and personal income were not associated with life satisfaction.

### Optimism

Overall, 74% of respondents thought that their life would be better in 1 year's time (Table 1).

Table 3 shows correlates of being optimistic in multilevel analysis. Odds of reporting optimism were 19% lower in those aged 20–24 than 15–19 years. Female participants had 43% higher odds of reporting optimism than male participants. Roma ethnicity and marital status were not significantly associated with optimism. Among those who had ever been married, marriage before age 18 years was not associated with optimism. Women who had children were 42% less likely to be optimistic than women without children.

Each quintile increase in household wealth was associated with a 13% increase in odds of optimism. Those residing in rural areas were 16% more likely to report optimism. Compared with primary level education, secondary education was associated with 49% greater odds of optimism and tertiary education with more than double the odds of optimism. Current studying was associated with 16% greater odds of optimism, and having a personal income with 22% higher odds of optimism. Current employment was not associated with optimism.

## Discussion

This analysis of MICS data revealed subjective well-being among representative samples of young people living in five Eastern European countries. Around 40% of young people considered themselves to be very happy or very satisfied with their life overall, while three quarters reported optimism. The five representative national samples, plus three Roma samples, varied in well-being measures and showed diverse demographic compositions. Multi-level analysis allowed the effect of country to be fixed while investigating individual-level correlates of well-being.

BiH and Ukraine demonstrated the lowest levels of subjective well-being of the five countries. As of 2012, these two countries also had the lowest GDP of the five countries (The World Bank Group, 2016), which is consistent with international patterns (Deaton, 2008; Levin *et al.* 2011). Within countries, wealth quintile was one of the strongest, most consistent predictors of subjective well-being. This is also consistent with existing research from other countries, much of which shows that relative wealth is a stronger predictor of well-being than absolute wealth (Dolan *et al.* 2008; Levin *et al.* 2011). While a certain level of wealth is needed for the fulfilment of basic needs, greater relative wealth may improve subjective well-being through positive social comparison (Diener *et al.* 1995).

There were key differences between Roma populations and non-Roma populations in terms of basic demographic characteristics. Particularly notable were the higher levels of marriage and childbearing, lower levels of educational attainment, and lower employment rates, particularly among Roma women. Despite this, there were no differences in levels of subjective well-being between Roma and non-Roma young people after controlling for other factors. This finding is in contrast to the only previous study of subjective well-being among Roma, which found that happiness and life satisfaction were poorer among Roma than non-Roma (Kamberi *et al.* 2015). The study, which included adults aged 16 to over 60 years, found that the difference in subjective well-being by ethnicity could be explained by the lower health status, lower income, lower education, lower quality of housing and greater perceived ethnic discrimination Roma experienced.

Employment was associated with life satisfaction, which is consistent with previous research (Dolan *et al.* 2008; Proctor *et al.* 2009). Despite high levels of educational attainment, just 20% of young women and 29% of young men reported being currently employed. Unemployment is a serious concern for young people in the region. The International Labour Organisation estimated a youth unemployment rate of 18% in 2013 in the Eastern European region (International Labour Organistion, 2014). Past research among adults has shown that in general Eastern Europeans report being less satisfied with their jobs and greater inequality in job satisfaction than Western Europeans (Borooah, 2009).

The proportions of young women who had children in the sample were high: 17% among non-Roma and 50% among Roma. For women, having children was associated with reduced optimism and life satisfaction. There is an extensive body of research demonstrating the negative impacts of early childbirth, including greater risk of maternal and child morbidity and mortality, pregnancy complications and worse outcomes for the child (Raj, 2010; Raj & Boehmer, 2013). This is the first study to show an impact of childbearing among young women on subjective well-being.

Current marriage was strongly associated with higher happiness and life satisfaction. Conversely, previous marriage (including widowing and divorce) and marriage at less than 18 years were associated with lower levels of subjective well-being. Previous research in adult populations has also found that married people are significantly happier (Stack & Eshleman, 1998; Diener *et al.* 2000; Dolan *et al.* 2008), but this is the first time a positive association between marriage and well-being has been shown among young people. This finding is noteworthy in the context of increasing age of marriage in economically developed countries; this trend may have consequences for well-being. On the other hand, those in our study who were married at <18 years reported being less happy than those who were married at 18 years and older. Child marriage (less than 18 years) was common among respondents (17% of those ever married in the general population and 64% among Roma). Previous research has also shown that child marriage is associated with multiple negative health outcomes (Raj, 2010; Raj & Boehmer, 2013).

Young people residing in rural areas demonstrated significantly higher levels of subjective well-being than those in urban areas, in keeping with findings from the USA and European Union countries (Berry & Okulicz-Kozaryn, 2011; Sorensen, 2014). Research from high-income countries shows that residential relocation correlates with lower life satisfaction (Proctor *et al.* 2009). We did not measure relocation, but urban youth may have relocated from rural areas for education and employment opportunities. Increased social cohesion and connection with nature have also been hypothesized as drivers of well-being in rural areas, though further research is needed on this topic (Sorensen, 2014).

Finally, younger age (15–19 years) was consistently associated with higher levels of subjective well-being than older age (20–24 years) after adjusting for other factors. A review of well-being research describes a U-shaped correlation between age and subjective well-being, with the youngest and oldest reporting greatest levels of subjective well-being (Dolan *et al.* 2008). Research with children and adolescents has mostly found a decline in subjective well-being at the onset of adolescence (Proctor *et al.* 2009) and the Gallup World Poll found an overall decline in life satisfaction with increasing age (Helliwell *et al.* 2015). In our study it is not clear if this reduction is an effect of ageing or an effect specific to these age cohorts. This uncertainty highlights the importance of longitudinal research with adolescents to determine the causes of changing well-being over the life course.

## Limitations

The cross-sectional nature of this study meant that we were unable to establish causation; correlations between demographic characteristics and subjective well-being may work both ways, as happy people, for example, may be more likely to succeed in schooling or get married. While the methodology controlled for variation between nationalities and demographic groups, other important confounding factors may have been neglected. For example, other factors previously associated with youth well-being that we did not measure include personality, attitudes, physical health, behaviour, social support, family functioning and life events (Proctor *et al.* 2009). Finally, there are aspects of subjective well-being, which have been measured in previous research, such as absence of negative affect, that were not included here (Diener *et al.* 2017).

## Conclusions

We identified large differences in young people's subjective well-being between different nationalities and different demographic groups. Multi-level, multivariable analysis allowed the identification of contextual factors consistently associated with higher levels of happiness, life satisfaction and optimism across the five countries, including younger age, current marriage, not having children, higher education, increased relative wealth and rural residence. Despite higher rates of adolescent marriage and childbearing, and lower levels of educational attainment and employment, Roma youth had similar levels of well-being to the general population.

Our findings provide further support for global health policies to reduce child marriage and delay parenthood for adolescent girls and women. In particular, these findings emphasize the important contribution of education to the well-being of young people. The finding that well-being and socio-economic status are strongly correlated even among adolescents and young adults deserves further study. Further research is also needed to better understand the association between rural residence and increased well-being in Eastern Europe, and to understand why well-being decreases with age between late adolescence and early adulthood.

This study highlights the value and feasibility of routinely collecting subjective well-being data in household surveys such as MICS. Continuing to collect these data from Eastern European young people and expanding to other regions will allow systematic comparison over time and space, giving insight into how political, societal and health system changes affect young people's well-being.
